# LINKING HEALTH ATTRIBUTES TO ACUTE-CARE USAGE IN SOUTH FLORIDA VETERANS, A PILOT SURVEY STUDY

**DOI:** 10.1093/geroni/igad104.3755

**Published:** 2023-12-21

**Authors:** Richard Munoz, Zoran Bursac, Carlos Gomez, Jared Hansen, Sandra Garcia, Diana Ruiz, Marianne Desir, Stuti Dang

**Affiliations:** Florida International University, Miami, Florida, United States; Florida International University, Miami, Florida, United States; Miami VA Healthcare System, Miami, Florida, United States; Veterans Affairs, Salt Lake City, Utah, United States; University of Miami, Miami, Florida, United States; Miami VA Healthcare System, Miami, Florida, United States; Miami VA Healthcare System, Miami, Florida, United States; University of Miami, Miami, Florida, United States

## Abstract

“High-Need, High-Risk” (HNHR) Veteran patients are identified quarterly by the Department of Veterans Affairs (VA) as having the top 5% predicted probability of hospitalization or mortality. We fielded a pilot survey to 2,543 older “HNHR” Veterans receiving care at the Miami VA Medical Center between October 2017 and September 2018, where 634 replied; mean age 70.61, standard deviation, 8.98, racially/ethnically White (403, 61.24%), Black (240, 36.47%), Hispanic (537, 81.61%) and nineteen women (3%). The survey contained 42 self-reported questions covering physical, mental, and social determinants of health, and access/use of healthcare resources. Two measured prior 6-month acute-care utilization outcomes: emergency room visits (ERV) and inpatient hospital admissions (IHA). Logistic regression was used to find health attributes associated with both outcomes, and latent class analysis was used to group Veterans into clinically relevant latent classes, analyzing health attributes endorsed by each. We found that transportation access issues to receiving healthcare were associated with both outcomes (OR = 2.980, p = 0.014), underscoring the role of social determinants of health. Self-perceptions of general health (OR = 0.806, p = 0.04), and a Veteran attending the Miami VA frailty clinic (OR = 0.435, p < 0.001), were associated with ERV and IHA, respectively. We identified four latent classes distinctly grouping patients by endorsing 1) depression; 2) not having a caregiver and being unmarried; 3) functional issues, and 4) being homebound and receiving home healthcare. These latent classes will help better inform clinical recommendations and program development to better meet needs for “HNHR” Veterans.

